# Experiences of Accessing Physical Rehabilitation Services and Assistive Products Among Orthosis and Wheelchair Users: Qualitative Study

**DOI:** 10.2196/84735

**Published:** 2026-07-29

**Authors:** Charlotte Spurway, Andrew Power, Maggie Donovan-Hall, Andrew Amos Channon

**Affiliations:** 1 Department of Applied Health Sciences College of Medicine and Health University of Birmingham Birmingham United Kingdom; 2 School of Geography and Environmental Science University of Southampton Southampton United Kingdom; 3 School of Health Sciences University of Southampton Southampton United Kingdom; 4 Department of Social Statistics and Demography University of Southampton Southampton United Kingdom

**Keywords:** Cambodia, orthotic devices, wheelchairs, physical rehabilitation, qualitative, resource-limited settings

## Abstract

**Background:**

Access to physical rehabilitation services, including orthotics and wheelchairs, is recognized as a fundamental human right but remains limited in many low- and middle-income countries due to resource constraints, fragmented care, and a shortage of trained rehabilitation professionals. In Cambodia, where the health system still faces challenges from past conflict, these services are delivered through a mix of government, international, and nongovernmental organizations.

**Objective:**

This study investigated the pathways to obtaining orthoses and wheelchairs in Cambodia and their impact on individuals with physical impairments.

**Methods:**

Semistructured interviews were conducted to explore experiences and the impact of accessing physical rehabilitation and assistive products for orthosis and wheelchair users. Interviews were conducted face-to-face with translation support, audio recorded, transcribed, and analyzed using thematic analysis. Interviews were conducted with participants in Phnom Penh and the Kandal province in Cambodia. In total, 17 participants aged 18 to 65 years who were current or previous patients of the Exceed Worldwide physical rehabilitation center located in Phnom Penh took part in the study.

**Results:**

Three themes emerged (early experiences of illness and health care seeking, pathways to assistive products, and the impact of these assistive products). Access to orthoses and wheelchairs often involved lengthy delays and inconsistent experiences with medical care and physiotherapy. Despite these challenges, assistive products played a vital role in improving mobility, independence, and social participation, contributing to greater confidence and emotional well-being. However, issues such as poor environmental accessibility and delays in repair or replacement limited their effectiveness.

**Conclusions:**

The findings demonstrate that, while assistive products offer significant benefits, they do not fully address the systemic barriers faced by people with physical disabilities in Cambodia. Persistent challenges such as stigma, environmental constraints, and emotional strain demonstrate the need for integrated policy reforms and a robust continuum of care that ensures coordinated, ongoing support across all stages of rehabilitation.

## Introduction

The United Nations Convention on the Rights of Persons With Disabilities states that access to physical rehabilitation services and assistive products constitutes a fundamental human right [1]. Consequently, individuals with disabilities must be guaranteed equitable access to these services. According to the World Health Organization [1], disability is caused by the interaction between health conditions or impairments and societal or environmental barriers that hinder full participation in society. Within this, impairment refers to issues in body function or structure, including significant deviations or losses. In many low- and middle-income countries (LMICs), physical rehabilitation centers providing prosthetic and orthotic (P&O) services and assistive products are limited, and those services that are available may be inaccessible to potential service users [2].

Assistive products, such as orthoses and wheelchairs, are external products that can be used to prevent impairments and can support individuals in many areas of life, such as education, employment, independent living, and social participation [1,3]. The provision of assistive products can bring socioeconomic benefits for governments and welfare systems, including increased labor force productivity [1,4]. Orthotics and wheelchairs help improve mobility, but they must be appropriate for the user’s environment. If an assistive product is not well matched to the setting in which it is used, it may be abandoned [2]. This can result from poor-quality provision, limited user training, and a lack of local services for maintenance and repair [2].

Although population need for wheelchairs and orthoses is lower than the need for other assistive products such as spectacles or walking sticks, reflecting their use among people with more severe functional limitations [1], access remains limited. Only around one-third of those needing basic manual wheelchairs have access to them, with even lower access to more specialized devices. Orthotic provision is similarly low, meeting only 15% to 25% of needs [1].

Orthoses and wheelchairs are included in the World Health Organization Priority Assistive Products List, reflecting their important role in enabling mobility, independence, and participation for large and diverse user groups [5]. However, access to assistive products in LMICs remains limited due to factors such as a lack of political prioritization, insufficient funding and investment, limited awareness of the need for assistive products, market barriers, and shortages of trained rehabilitation workers, resulting in understaffed services and barriers to effective service delivery [6,7]. These challenges can contribute to lengthy delays in accessing physical rehabilitation, such as P&O services, which are further exacerbated by limited referral systems in place and the absence of well-established continuums of care across multiple stages, such as health promotion, preventive care, curative care, and rehabilitation [1,6,8].

This research focused on Cambodia, an LMIC in Southeast Asia. The health system in Cambodia has been heavily impacted by a turbulent past, including conflict and civil war, which has contributed to ongoing challenges [9]. Currently, the delivery of physical rehabilitation services in Cambodia is a joint effort among the Cambodian government, international organizations, and nongovernmental organizations (NGOs) [10]. The People With Disabilities Foundation, a department of the Cambodian Ministry of Health, operates 3 physical rehabilitation clinics and 2 repair workshops [10]. Other organizations, such as Exceed Worldwide, International Committee of the Red Cross, and Humanity & Inclusion, also provide physical rehabilitation and assistive products free of charge [11].

Although there is a growing body of qualitative research on prosthetic services in low‑resource settings, including studies on access barriers [12,13], user experiences [11,14], and satisfaction with devices and service delivery [15], fewer studies focus specifically on orthosis or wheelchair users. Where included, these groups are typically examined alongside prosthetic users, limiting evidence on their specific experiences despite the scale and diversity of people who rely on these devices. This study included wheelchair users only, orthosis users only, and users of both to explore shared and divergent experiences across mobility-related assistive product use, enabling analysis of experiences related to rehabilitation access, assistive technology provision, participation, and disability-related stigma, as well as differences between groups. This study therefore explored pathways to accessing physical rehabilitation and obtaining orthoses and wheelchairs in Cambodia and examined how this access shapes the lives of people with physical impairments.

## Methods

### Design

This qualitative study used semistructured in-depth interviews to explore the pathways to physical rehabilitation services for orthotics and wheelchairs in Cambodia. As this study aimed to understand the pathways taken to receive services, we wanted participants to use their own words to describe their experiences rather than using a fixed survey, leading to the approach undertaken. Multimedia Appendices 1 and 2 contain the completed COREQ (Consolidated Criteria for Reporting Qualitative Research) checklist and associated information about the interviewer.

### Setting and Sampling

This study took place in Phnom Penh and the Kandal province, Cambodia, in partnership with a physical rehabilitation center operated by Exceed [16]. Exceed is an international NGO that provides physical rehabilitation services in 3 provinces of Cambodia. Participants included individuals who were currently using or had previously received an orthosis or wheelchair from Exceed’s Phnom Penh clinic.

Participants were primarily recruited using a convenience sampling approach involving individuals who attended the clinic either for scheduled appointments or a walk-in visit. Additional recruitment took place during community visits conducted as part of Exceed’s outreach activities, including repair workshops and wheelchair delivery events. Some participants were also identified through direct outreach, in which community workers contacted potential participants to inquire about their interest in taking part in the study. A total of 9 participants were recruited at the clinic, and 8 were recruited either during community visits or through interviews that took place outside of the clinic arranged by community workers. Recruitment was conducted by Exceed staff, who presented the study and emphasized that participation was voluntary.

### Interview Procedures

All interviews were conducted face-to-face in Cambodia at Exceed's clinic in Phnom Penh, during community outreach visits or at the homes of participants. For interviews conducted during community visits, a private area within the community space where outreach activities were held was used to ensure confidentiality. The interviews took place from April 2022 to June 2022.

A lecturer from the Cambodian School of Prosthetics and Orthotics in Phnom Penh served as the translator for the interviews. The translator was a native Khmer speaker with English as their second language and professional experience teaching P&O in English. Their clinical and educational expertise supported the translation of technical rehabilitation concepts; however, the absence of a professionally trained interpreter may have resulted in the loss of linguistic nuance or subtle shifts in meaning [17]. Consistent use of a single translator and advance translation of the interview guide were used to support translation consistency [18]. The English version of the interview guide can be found in Multimedia Appendix 3.

During the interviews, questions asked in English by the interviewer were translated into Khmer. Participant responses were then translated back into English using consecutive verbatim interpretation. The quotations presented in the Results section represent the words of the translator and were subsequently edited into readable English for clarity while preserving the original semantic meaning and tone, in line with recommendations within the qualitative research literature regarding translated data [19,20].

The interviews were also recorded using an audio device. Sample size was guided by the aim of generating in-depth accounts rather than achieving data saturation, in line with reflexive thematic analysis practice [21].

### Ethical Considerations

All participant documents, such as participant information sheets and consent forms, were produced in English and translated into Khmer by a local team based at Exceed. These documents were provided to the participants before the interviews, and the translator read them to participants to ensure full agreement and explained that they could withdraw at any time and this would not impact the care or treatment they received from the P&O clinic. The potential risks and benefits of taking part and data handling procedures were also explained to participants.

Once the participants had understood and agreed to take part in the study, written informed consent was obtained from all participants prior to data collection. No participants withdrew from the study following consent. All identifiable information was removed from transcripts, and data were stored securely on university servers accessible only to members of the research team.

Ethics approval for this study was granted by the University of Southampton Ethics and Research Governance Online system (68254.A1) and the National Ethics Committee for Health Research in Cambodia (088). All study procedures were conducted in accordance with the ethical standards of the approving committee. Participants received a 12,000 Cambodian riel (US $2.97) token of appreciation for their time.

### Data Analysis

The audio files from the interviews were transcribed verbatim by the lead author (CS). The data were analyzed using reflexive thematic analysis [21] to categorize them into themes and subthemes. Thematic analysis was carried out using an interpretivist epistemological framework, which views knowledge as coconstructed and embedded within social, cultural, and contextual settings [22]. From this perspective, findings are not considered objective truths but interpretations shaped through interaction between translated participant accounts and researcher analysis.

Data coding was completed by the lead author (CS) and discussed with the coauthors (AP, MD-H, and AAC). This study formed part of a wider project on barriers to and facilitators of health and rehabilitation services in Cambodia. The wider study informed the initial deductive coding framework; however, coding and theme development for this analysis were primarily inductive and grounded in participants’ accounts. The wider study provided a guiding concept rather than a rigid coding structure, allowing additional meanings and interpretations to develop iteratively throughout the analysis through inductive coding [23]. After coding the interview data, codes were reviewed and iteratively developed into broader themes and subthemes through an ongoing process of comparison, refinement, and discussion among the research team. Multimedia Appendix 4 includes all the themes, subthemes, and codes that emerged in this study.

## Results

### Participant Characteristics

Table 1 presents participant information, including gender, age, type of assistive product, reason for use, and interview location. Seventeen participants were interviewed (n=12, 70.6% women and n=5, 29.4% men), and none dropped out before completing the interview. In total, 52.9% (n=9) of the participants used knee-ankle-foot orthoses, 29.4% (n=5) used ankle-foot orthoses, and 17.6% (n=3) used manual wheelchairs. The average interview length was 43 (SD 12.3) minutes (ranging from 19 to 62 minutes).

**Table 1:** Participant information.

Pseudonym	Sex	Age (y)	Device	Reason for device use	Location of interview
Bopha	Female	40	KAFO^a^	Polio	Clinic
Sambath	Male	31	AFO^b^	Polio	Clinic
Chaya	Female	55	KAFO	Polio	Clinic
Chhean	Female	30	KAFO	Polio	Clinic
Daevy	Female	33	AFO	Polio	Clinic
Leap	Female	35	AFO	Polio	Community
Sothy	Female	38	KAFO	Polio	Clinic
Samnang	Female	18	AFO	Cerebral palsy	Clinic
Sophal	Female	51	KAFO	Polio	Community
Chamroeun	Female	20	AFO	Cerebral palsy	Community
Rotha	Female	18	KAFO	Polio	Clinic
Choun	Female	18	KAFO	Clubfoot	Clinic
Chea	Female	28	Wheelchair	Spinal condition	Community
Narin	Male	65	Wheelchair	Stroke	Community
Chanthou	Male	51	Wheelchair	Polio	Community
Savy	Male	35	KAFO	Polio	Community
Viseth	Male	32	KAFO	Polio	Community

^a^KAFO: knee-ankle-foot orthosis.

^b^AFO: ankle-foot orthosis.

The interviews were coded into three themes: (1) early experiences of illness and health care seeking, (2) pathways to assistive products, and (3) impact of assistive products.

### Early Experiences of Illness and Health Care Seeking

Most participants stated that the reason for orthosis or wheelchair use was fever, with the most common cause of this being polio. Nearly all participants experienced this before they were 5 years old. A minority were uncertain of the cause, with some of their parents being skeptical of medical explanations. For example, one participant reported that her mother believed that antibiotic or antipyretic injections received at a health center were responsible:

Before she got ill, she was able to walk right, so her mum doesn’t believe that a fever for a night has led to her being unable to stand and walk.

From interview with Samnang; female; aged 18 years

As most participants were young at the time of diagnosis, health care decisions were made by parents and grandparents. Health care use varied; while some sought multiple treatments, others received no care despite sudden limb weakness or paralysis. Participants who reported never accessing health care at the time cited several reasons, including parental work demands, financial constraints, and limited health care availability. This was explained by 11.8% (2/17) of the participants, who described the lack of treatment options when they were children due to insecurity and civil unrest in the country:

During 1972, she said because the country is insecure or there were problems in the country, there was no medical treatment available, no nothing.

From interview with Sophal; female; aged 51 years

Khmer traditional treatments were used exclusively or alongside allopathic medicine, with parents often moving between different types of health care facilities.

### Pathways to Assistive Products

#### Experiences of Medical Treatments or Physiotherapy

Participants learned of orthotic and wheelchair services, including those provided by Exceed and other facilities, through family, neighbors, and outreach by community health workers. Lengthy delays between the onset of impairment and receiving further medical treatment, such as surgery or physiotherapy from hospitals or physical rehabilitation services, were common. Surgery, typically to release lower-limb muscles and tendons to improve mobility, was followed by months of aftercare, although some participants were unable to adhere to it. One participant explained that it was recommended that she undergo surgery on her spine; however, her mother refused due to not believing it was necessary:

The physician in Kantha Bopha hospital mentioned that the most important thing is her trunk, her spine. They wanted to do the operation on her spine, but her mum didn’t agree with the surgery.

From interview with Chea; female; aged 28 years

Although it was not explored in depth, these findings indicate the importance of parental trust in obtaining health care and ensuring that health care professionals can adequately explain information to foster trust between them and patients.

#### Experiences of Using P&O Services

Participants reported varied experiences with physical rehabilitation services and assistive products. Initial visits typically involved assessment for orthotics or wheelchairs followed by physiotherapy and/or limb casting for orthotic fabrication. Several participants recalled fear and distress during early fittings, particularly as children, due to limited understanding of the process. One participant, who was a child at the time of her first appointment, stated that she believed the orthosis would require cutting into her leg:

She said that first time she was scared because she thought that when they brought her to the center, they might cut into her leg or something.

From interview with Daevy; female; aged 35 years

Two wheelchair users reported no difficulties adapting; however, one wheelchair user expressed reluctance to use the wheelchair outside the home:

He uses the wheelchair just around the house, only indoors. He doesn’t use it outdoors because he is afraid of cars and other transport, because they travel very fast, so he is worried about accidents.

From interview with Narin; male; aged 65 years

Pain during orthosis fitting and use was commonly reported, particularly by those fitted as children. Gender differences emerged, with men generally reporting minimal discomfort whereas women more often described pain and reported their orthoses as bulky, heavy, and causing blisters in the lower limbs. The 2 quotes below describe some of the experiences that female participants had with their orthoses:

She said that when using the device, she feels like it’s difficult to walk and is painful. Sometimes she feels like the knee is soft, so she falls over often.

From interview with Rotha; female; aged 18 years

He said it’s not very difficult, like he is able to adapt when he wears the orthosis.

From interview with Sambath; male; aged 31 years

Difficulties in adapting to an orthosis sometimes led to device abandonment, particularly when discomfort prevented use. However, many users persisted because they recognized potential functional benefits, although some required multiple orthoses to adapt:

She said that after she threw the first device away, she was afraid she would be shy (due to her impairment), so she wanted to try and get a second device and learn to walk with that device.

From interview with Bopha; female; aged 40 years

### Impact of Assistive Products

#### Importance of Access to Assistive Products

Several participants reported the positive impact of using a wheelchair or an orthosis. These devices supported their mobility, independence, and inclusion within their communities. Access to assistive products improved participants’ confidence and emotional well-being, contributing to a greater sense of happiness and facilitating social interaction. One participant explained that, since she received the orthotic device, she had been able to make friends and work with others:

She feels confident and can be herself when she got the new device, before when she didn’t have the device, she didn’t have friends and worked alone.

From interview with Chhean; female; aged 30 years

Independence and the ability to carry out household tasks were highly valued, influencing patterns of assistive product use. Orthoses were generally worn throughout the day and removed only for washing or sleep, although some participants managed short distances without them. This pattern of use contrasted with that of wheelchairs, which were less commonly used within the home environment:

For him if he just stays at home, he won’t use the wheelchair, but if he travels outside for a short distance, he’s going to use his wheelchair.

From interview with Chanthou; male; aged 51 years

Despite the significance of assistive products in participants’ lives, many reported delays in repairs or replacements of their assistive products. One participant, for example, reported using the same orthotic device for over 10 years, far beyond the typical expected life span of 2 to 3 years. These delays often led participants to resort to temporary self-repairs:

He said that his orthosis was broken, so he tried to repair by wrapping plastic around it, but he knows that it is not strong and is only a temporary repair until he gets the new device.

From interview with Sambath; male; aged 31 years

#### Limits in Access to Assistive Products

While assistive products provided significant benefits, they did not eliminate all challenges associated with having a physical impairment in Cambodia. One participant noted that, after discontinuing the use of her orthosis, she felt that her lower limbs had become stronger and became more confident in her mobility:

She said that because she has stopped using the device, she feels stronger and more confident because when she was young, she fell over often, but now it’s ok, she doesn’t fall often.

From interview with Chamroeun; female; aged 20 years

Participants noted that environmental conditions affected the functionality of their assistive products. Uneven or sandy terrain increased the risk of falls and wheelchair entrapment, whereas the weather reduced durability and comfort. Flooding and heavy rains during the wet season caused rusting of wheelchair frames, whereas high temperatures affected orthoses by causing the plastic to expand, affecting the fit of the device:

Because of the heat right now, the plastic [on the orthosis] increases in size so it makes it loose for him. It doesn’t cause pain, but there is less control.

From interview with Savy; male; aged 35 years

Despite access to assistive products, daily limitations persisted, varying across individuals. Participants reported avoiding community events such as weddings and religious ceremonies due to discomfort; anxiety; or practical barriers, for example, concerns about not being able to sit on the floor (customary during temple visits) without removing an orthosis.

The interviews also found challenges with self-perception. Several participants expressed feelings of being a burden on their families due to their reliance on others. One participant, following a stroke, compared his current abilities with his prestroke abilities and described a reduced sense of self:

The people around him pity him. He feels like before he could do everything and worked hard, but now he can’t do anything, so he feels like he is useless.

From interview with Narin; male; aged 65 years

Experiences of negative perceptions were common. Some participants reported being stared at in public, leading to a reluctance to leave their homes:

Other problems he faces when he goes out, people look at him like he is not normal and they stare at him.

From interview with Chanthou; male; aged 51 years

However, not all participants reported discrimination. Some felt that they were treated equally because they were able to perform daily tasks and contribute economically, aligning with societal perceptions of “normality”:

She doesn’t feel like anyone discriminates or criticizes her about her disability. Her family say that she is ok, she has an impairment, but she is able to get married, have a family and work like other normal people.

From interview with Leap; female; aged 38 years

Participants often described employment and contributing to family and community life as helping mitigate the impact of impairment. “Blending in” with people without disabilities was seen as a strategy to avoid discrimination. Some noted improving attitudes toward disability in Cambodia, attributed to greater awareness and education:

Nowadays he thinks that information about disability is shared to everyone and people understand more about disabled people, so they don’t discriminate against him.

From interview with Viseth; male; aged 32 years

However, there was a shared recognition of the ongoing need for more information about disability to be disseminated through schools, television, and other media to reduce stigma.

## Discussion

### Principal Findings

This study found that early childhood illness, most commonly linked to polio, alongside delayed or absent access to health care and rehabilitation services, shaped long-term disability trajectories and pathways to assistive product use. Assistive products substantially improved mobility, independence, and psychosocial well-being but did not fully remove environmental, social, and attitudinal barriers experienced by users. Pain, fear, and adaptation challenges highlighted the limitations of provision models that focus solely on the device, emphasizing the need for more holistic approaches that address both individual needs and the wider social context.

These broader challenges are rooted in the underlying causes and understandings of impairment. Use of orthoses or wheelchairs in this study was largely attributed to contracting polio in childhood, although other causes included clubfoot, cerebral palsy, and stroke. Some participants with polio were uncertain about their exact diagnosis, with several attributing their impairments to treatments received in hospital. Evidence shows that, in polio‑endemic settings, intramuscular injections administered during the incubation period of poliovirus can increase the risk of paralysis, a phenomenon known as provocation poliomyelitis [24-26]. This may help explain why some individuals linked their impairments to medical treatments they received as children.

Many participants reported significant delays in receiving their first assistive product, primarily due to limited awareness of P&O services and restricted service availability. Early rehabilitation is known to improve outcomes for conditions such as stroke and cerebral palsy [27,28]. However, access to physical rehabilitation in many LMIC settings remains constrained by inconsistent service provision and shortages of skilled rehabilitation professionals [29]. Pathways to assistive products can also be difficult to navigate due to fragmented referral systems, particularly where rehabilitation services are delivered by international organizations and NGOs operating separately from wider health systems [1]. Despite this, P&O and other rehabilitation services are essential components of the health care system and play an important role along the continuum of care, particularly in ensuring access during both the postacute and long-term phases of care [30].

Initial use of orthoses was frequently associated with pain, contributing to device abandonment in some cases. This study found that female users of orthotics described experiences of pain and discomfort with their orthoses more frequently than male users. This aligns with existing literature that showed that women’s experiences with orthoses were more unpleasant and uncomfortable [31], with another study finding persistent, gendered disparities in access, design, comfort, and function outcomes of assistive products for women compared to men [32]. An analysis of data on orthosis users in Cambodia found that the proportion of current female users was nearly 10% lower than that of male users [33]. This highlights the need for more research specifically examining gendered outcomes of orthoses and wheelchairs. Addressing these gaps will require approaches that account for physiological differences, user experience, and the structural factors shaping women’s rehabilitation outcomes [34].

The findings from this study highlight the role of assistive products in the lives of people with physical impairments. Participants reported that these products enhanced independence, enabled work, and supported daily activities, echoing findings from other studies involving P&O users [11,35]. However, access alone does not ensure use, nor do assistive products fully remove barriers, especially in contexts where the broader social and political environment remains restrictive or stigmatizing [3,36]. In such settings, ongoing barriers to education and employment may constrain the potential of assistive devices to advance inclusion.

Experiences of discrimination or prejudice related to physical disabilities were reported by participants. Misconceptions about the cause of disability, often rooted in traditional or religious understandings, can help perpetuate disability stigma [37,38]. Such stigma can restrict access to employment, education, and community inclusion. However, not all participants experienced discrimination or identified as having a disability, and some described their ability to work and have a family as being the same as that of others. Additionally, it was reported that wearing an assistive product reduced negative treatment from others. Previous research has shown that access to resources that can be used to obtain assistive products, such as finances and knowledge, was an indicator of privilege [39]. This finding was linked to the notion that poverty stigma can be more discriminatory than disability stigma in LMICs. This may help explain why participants had more positive attitudes after receiving an orthosis or wheelchair.

Not all participants wore an orthotic device daily or at all, underlining that access alone does not guarantee use of assistive products or rehabilitation services. In many LMICs, limited design options may not suit users’ environments, contributing to nonuse [1,2]. While this study did not examine device abandonment in depth, findings suggest that provided products may not always meet users’ needs or preferences. Known contributors to abandonment include pain, discomfort, inadequate training, and personal preference [40,41]. These gaps highlight the need for a comprehensive service approach that goes beyond initial provision to include follow-up, context-appropriate design, user education, and sustained support across the continuum of care.

In Cambodia, P&O service provision has shifted over time, with changes in rehabilitation services, increased government involvement, and reduced international funding. Sustaining a continuum of care for those requiring physical rehabilitation depends on establishing standardized referral pathways across all levels of the health system [30]. This necessitates collaboration among a range of stakeholders, including the government, public and private hospitals and health facilities, and international organizations.

### Reflexivity

As White, anglophone researchers based in a high-income country, we recognize that our professional backgrounds, experiences, and prior assumptions may have shaped the study design, data interpretation, and construction of meaning from the data. The absence of Cambodian researchers within the author team is an important limitation and reflects broader structural inequities in global health research and ongoing debates around decolonizing humanitarian and development work. This study was conducted as part of a PhD project, which influenced the research team composition and authorship structure and may have limited the extent to which locally grounded interpretations informed the analysis.

Data collection and interpretation were supported by Cambodian rehabilitation professionals who facilitated recruitment, translation, and contextual understanding. Although their contributions informed the research process and were remunerated, they were not involved in coauthorship or full thematic analysis, primarily due to limited availability alongside clinic responsibilities. This may have influenced how meaning was interpreted and themes were developed.

The interviewer, as an external researcher, also occupied an outsider position [42], which may have shaped participant responses and the coconstruction of interview data. To enhance reflexivity and minimize bias, the findings were discussed within the research team, and contextual input from in-country collaborators informed interpretation. Nevertheless, limited local analytical involvement remains a key limitation, and future research should prioritize more equitable coproduction, including shared analysis and authorship with Cambodian researchers.

### Strengths and Limitations

This study has a number of strengths. First, it makes a unique contribution to the literature as there is limited empirical research examining access to physical rehabilitation services specifically among individuals who use orthoses and wheelchairs. While there is a growing body of work on assistive technology access in LMICs, most studies focus on prosthetics or broader disability populations, with relatively few exploring the distinct needs and barriers experienced by orthosis and wheelchair users. Second, the findings are situated within the social, cultural, and environmental contexts of participants. This contextual grounding is important in rehabilitation and assistive technology research as cultural perceptions of disability, social support networks, and environmental barriers shape access, use, and outcomes [1].

This study also has some limitations (beyond those noted above about reflexivity). Participants were exclusively current or former users of Exceed services in Phnom Penh, excluding those who accessed other Exceed centers or services from different rehabilitation organizations, who may have had different pathways to obtaining orthoses and wheelchairs.

The interviewer (CS) did not speak Khmer, requiring the use of a translator. While clarification was sought to ensure accurate interpretation, some loss of meaning may have occurred. The translations were conducted by a local colleague, who is a native speaker of Khmer without formal training in professional interpretation or translation. This is a limitation of the study as translation in qualitative research is an interpretive act and can influence the precision and nuance of participant meaning [17]. To mitigate this, the same translator was used throughout the study to support consistency, and the interview guide was translated in advance to reduce ad hoc interpretation during interviews [18]. The translator also had contextual familiarity with the study setting, which supported comprehension of local and physical rehabilitation terminology. However, it is recognized that these measures do not replace the role of a professionally trained interpreter and that limited formal linguistic training may have influenced the accuracy and richness of some translations. The translator's role at the Cambodian School of Prosthetics and Orthotics in Phnom Penh, which collaborates closely with Exceed, may have also influenced participants’ willingness to voice criticisms, although assurances were given that care would not be affected.

### Conclusions

This study investigated the pathways to physical rehabilitation services for users of orthoses and wheelchairs in Cambodia. The experiences of participants offer valuable insights into how individuals find and engage with P&O services for physical rehabilitation and assistive products and how these interventions influence their mobility and livelihood. This study highlights the important role of assistive products in enhancing mobility, independence, and engagement in daily activities. However, it also shows that they cannot fully overcome broader environmental, social, and emotional barriers, including stigma, discrimination, and negative self-perception.

Assistive products must be part of a broader, continuous system of care. A functioning continuum of care requires standardized referral mechanisms, consistent follow-up, and integration across all levels of the health system. Without this, individuals risk falling through gaps in service provision, particularly during critical stages of life such as childhood and adolescence.

Future research should further investigate the causes of assistive product abandonment and explore the complex reasons why individuals may choose not to use assistive products. A holistic, person-centered approach, one that supports individuals across the full continuum of care and acknowledges both physical and sociocultural dimensions, is key for improving quality of life for people with physical impairments in Cambodia.

## Data Availability

The datasets generated and analyzed during this study are publicly available in the University of Southampton institutional research repository [43]. The repository contains the deidentified study data and accompanying documentation required to reproduce the analyses.

## Appendix

Multimedia Appendix 1 COREQ checklist.

Multimedia Appendix 2 Additional reflexivity statement.

Multimedia Appendix 3 Interview guide.

Multimedia Appendix 4 Themes and codes.

## Abbreviations

COREQ: Consolidated Criteria for Reporting Qualitative Research
LMIC: low- and middle-income country
NGO: nongovernmental organization
P&O: prosthetic and orthotic
