# Supplementary material for: Experiences of Accessing Physical Rehabilitation Services and Assistive Products Among Orthosis and Wheelchair Users: Qualitative Study
Source: JMIR Rehabil Assist Technol. 2026 Jul 29;13:e84735. doi: 10.2196/84735 (PMC13469828; doi:10.2196/84735)
Supplement: Supplementary file 1 [file rehab_v13i1e84735_app1.docx]

**COREQ (COnsolidated criteria for REporting Qualitative research) Checklist**

| **Topic** | **Item No.** | **Guide Questions/Description** | **Reported on Page No** |
| --- | --- | --- | --- |
| **Domain 1: Research team and reflexivity** | | |  |
| *Personal characteristics* | | |  |
| Interviewer/facilitator | 1 | Which author/s conducted the interview or focus group? | Multimedia appendix 2 |
| Credentials | 2 | What were the researcher’s credentials? E.g. PhD, MD | Multimedia appendix 2 |
| Occupation | 3 | What was their occupation at the time of the study? | Multimedia appendix 2 |
| Gender | 4 | Was the researcher male or female? | Multimedia appendix 2 |
| Experience and training | 5 | What experience or training did the researcher have? | Multimedia appendix 2 |
| *Relationship with participants* | | |  |
| Relationship established | 6 | Was a relationship established prior to study commencement? | Multimedia appendix 2 |
| Participant knowledge of the interviewer | 7 | What did the participants know about the researcher? e.g. personal goals, reasons for doing the research | Multimedia appendix 2 |
| Interviewer characteristics | 8 | What characteristics were reported about the inter viewer/facilitator? e.g. Bias, assumptions, reasons and interests in the research topic | Multimedia appendix 2 |
| **Domain 2: Study design** | | |  |
| *Theoretical framework* | | |  |
| Methodological orientation and Theory | 9 | What methodological orientation was stated to underpin the study? e.g. grounded theory, discourse analysis, ethnography, phenomenology, content analysis | 3 |
| *Participant selection* | | |  |
| Sampling | 10 | How were participants selected? e.g. purposive, convenience, consecutive, snowball | 2-3 |
| Method of approach | 11 | How were participants approached? e.g. face-to-face, telephone, mail, email | 2-3 |
| Sample size | 12 | How many participants were in the study? | 3 |
| Non-participation | 13 | How many people refused to participate or dropped out? Reasons? | 3 |
| *Setting* | | |  |
| Setting of data collection | 14 | Where was the data collected? e.g. home, clinic, workplace | 2-3 |
| Presence of non-participants | 15 | Was anyone else present besides the participants and researchers? | 3 |
| Description of sample | 16 | What are the important characteristics of the sample? e.g. demographic data, date | 3-4 |
| *Data collection* | | |  |
| Interview guide | 17 | Were questions, prompts, guides provided by the authors? Was it pilot tested? | 3 |
| Repeat interviews | 18 | Were repeat interviews carried out? If yes, how many? | N/A |
| Audio/visual recording | 19 | Did the research use audio or visual recording to collect the data? | 3 |
| Field notes | 20 | Were field notes made during and/or after the interview or focus group? | N/A |
| Duration | 21 | What was the duration of the interviews or focus group? | 3 |
| Data saturation | 22 | Was data saturation discussed? | 3 |
| Transcripts returned | 23 | Were transcripts returned to participants for comment and/or | N/A |

Developed from: Tong A, Sainsbury P, Craig J. Consolidated criteria for reporting qualitative research (COREQ): a 32-item checklist for interviews and focus groups. *International Journal for Quality in Health Care*. 2007. Volume 19, Number 6: pp. 349 – 357
