# Supplementary material for: Experiences of Accessing Physical Rehabilitation Services and Assistive Products Among Orthosis and Wheelchair Users: Qualitative Study
Source: JMIR Rehabil Assist Technol. 2026 Jul 29;13:e84735. doi: 10.2196/84735 (PMC13469828; doi:10.2196/84735)
Supplement: Supplementary file 2 [file rehab_v13i1e84735_app2.docx]

**Reflexivity statement**

At the time of the interviews, the lead author was undertaking her PhD at the University of Southampton, having already completed a BSc and MSc. Her doctoral research focused on access to health and rehabilitation services in Cambodia for people with physical disabilities. She is a white female researcher from the UK. The interviewer had received training in qualitative research methods and was supported by co-authors with extensive experience in qualitative methods and cross-cultural interviewing. The researcher was not known to participants beforehand. She was introduced to participants through a translator, who explained her name, role, and background, as well as providing information about the study, explaining the Participant Information Sheet in Khmer, and giving participants a Khmer-language copy of the study documents.
