# Supplementary material for: Experiences of Accessing Physical Rehabilitation Services and Assistive Products Among Orthosis and Wheelchair Users: Qualitative Study
Source: JMIR Rehabil Assist Technol. 2026 Jul 29;13:e84735. doi: 10.2196/84735 (PMC13469828; doi:10.2196/84735)
Supplement: Supplementary file 3 [file rehab_v13i1e84735_app3.docx]

**Interview guide**

| **Theme** | **Main questions** | **Probing questions/phrases** |
| --- | --- | --- |
| Background | - Please tell me a bit about yourself, for example you age, schooling and occupation. - How many people are in your household and what is your relationship to them? |  |
| Disability/ condition | I would like to hear more about your disability/ diagnosis.   - Please could you describe your condition to me in as much detail as you would like? - When or how did the condition develop/ occur? | - How does your disability/ condition impact you? This could be everyday life, work, schooling. - How does it make you feel? |
| Care seeking behaviour – including orthotics | - When did you first seek healthcare for your condition? Including hospitals and health centres, not just P&O services - When did you seek orthotic services? - Was the Exceed Worldwide clinic you have most recently attended the first clinic that your attended? - Have you attended any other rehabilitation centres? Including those not operated by Exceed Worldwide. - How did you hear about the orthotic services? | - Was this a decision made solely by you, or also by family members? - Where are all the places you sought care? - How was your experience? How did it make you feel? - Were you referred by a doctor/ medical professional? |
| Impact of orthotic | - What impact has the orthotic device had on your everyday life? - Do people treat you differently now you have your orthoses, compared to your life without it? - Has your orthotic device had an impact on your quality of life? | - Work, schooling, community participation… |
| Decision-making process | - Thinking of your most recent visit, what did you attend the clinic for? - How long did you wait before coming to the clinic? - Were there any barriers preventing you from attending the clinic sooner? - Is this clinic closest to your home? | - Did you require a repair or delivery of new orthotic device? - How long did it take you to travel to the clinic today? - If this is not the closest clinic to your home, what made you decide to come to this clinic, instead of the one closest? |
| Barriers to seeking orthotics | - If any, what barriers to seeking and utilising orthotic services have you experienced? - Does the environment around you support or hinder the utilising rehabilitative services for your condition? - What have the clinic done to mitigate the barriers to seeking care? | - Geographic barriers - Cultural barriers |
| Suggestions for P&O/ disability services | - In your opinions, what should the family, community, and the government do for the people who use orthotics or have disabilities impacting their mobility? | - Family? - Community? - Government? |
| Closing | - Is there anything else that you would like to add that I didn’t ask you? |  |
