# Supplementary material for: Experiences of Accessing Physical Rehabilitation Services and Assistive Products Among Orthosis and Wheelchair Users: Qualitative Study
Source: JMIR Rehabil Assist Technol. 2026 Jul 29;13:e84735. doi: 10.2196/84735 (PMC13469828; doi:10.2196/84735)
Supplement: Supplementary file 4 [file rehab_v13i1e84735_app4.docx]

**Themes and code generated from the interview data**

| **Themes** | **Theme description** | **Sub-themes** | **Codes** | **Code description** |
| --- | --- | --- | --- | --- |
| Early Experiences of Illness and Healthcare Seeking | This theme explores the medical conditions leading to orthosis or wheelchair use and participants’ experiences of seeking and accessing healthcare. | N/A | Cause of impairment | Understanding of the underlying medical condition leading to orthosis or wheelchair use; participants’ awareness of reasons for device prescription. |
|  |  |  | Impairment attributed to treatment | Belief that impairment was caused by medical interventions (e.g., injections or treatments received in health facilities). |
|  |  |  | Use of formal healthcare | Engagement with allopathic healthcare services to address their condition. |
|  |  |  | Khmer traditional  treatments | Seeking traditional medicine as an alternative or complement to formal healthcare. |
|  |  |  | Reasons for not seeking healthcare | Explanations for delays or avoidance of healthcare access. |
| Pathways to assistive products | This theme examines participants’ experiences with prosthetics and orthotics (P&O) services, including rehabilitation, referral routes, and adaptation to devices. | Experiences of medical treatments / physiotherapy | Physical therapy | Receiving physiotherapy before using an assistive product. |
|  |  |  | Hospital treatments | Undergoing hospital-based care before accessing assistive products. |
|  |  | Experiences of using P&O services. | Delays in accessing P&O services | Long waiting times before receiving orthoses or wheelchairs. |
|  |  |  | Initial experiences of P&O services | First encounters with assistive product provision. |
|  |  |  | Difficulties adapting to devices | Pain, discomfort, or fear when first using orthoses or wheelchairs. |
|  |  |  | Device abandonment | Discontinuing use of the first assistive product received. |
|  |  |  | Successful adaptation | No difficulties in adjusting to the device and sustained use |
| Impact of assistive products | This theme captures the positive and negative consequences of using assistive products, including physical, social, and psychological impacts. | Importance of access to assistive products | Positive emotions and confidence | Feelings of happiness, confidence, and improved wellbeing following device use. |
|  |  |  | Enhanced independence | Ability to perform daily tasks such as housework, employment, and self-care. |
|  |  |  | Improved social treatment | Better treatment from others after beginning device use. |
|  |  |  | Consistent use | Wearing or using the assistive product throughout the day. |
|  |  |  | Self-repairs | Making personal repairs to orthoses or wheelchairs rather than relying on P&O services. |
|  |  | Limits of access to assistive products | Impact of weather | Difficulties in device use during hot, rainy, or humid conditions. |
|  |  |  | Partial or inconsistent use | Reports of limited or selective use of devices. |
|  |  |  | Impact of impairment on daily life | Restrictions on participation in social and community events and limitations in carrying out household tasks, fetching water, or maintaining independence |
|  |  |  | Psychological and emotional impacts | Feelings of depression and reduced self-esteem linked to impairment. Experiences of thoughts about suicide. |
|  |  |  | Experiences of stigma and discrimination | Bullying, teasing, staring, or judgement by others. |
|  |  |  | No experiences of discrimination | Perceptions of little to no negative social treatment. |
|  |  |  | Disability awareness and education | Increased awareness of disability compared to previous years. Ongoing perception that disability remains a marginalised or under-discussed issue. |
